# Characteristics and Outcome of Vascular Graft Infections: A Risk Factor and Survival Analysis

**DOI:** 10.1093/ofid/ofae271

**Published:** 2024-05-13

**Authors:** Leonie Stockschläder, Donara Margaryan, Safwan Omran, Martin Schomaker, Andreas Greiner, Andrej Trampuz

**Affiliations:** Center for Musculoskeletal Surgery, Charité–Universitätsmedizin Berlin, Corporate Member of Freie Universität Berlin, Humboldt-Universität zu Berlin, and Berlin Institute of Health, Berlin, Germany; Center for Musculoskeletal Surgery, Charité–Universitätsmedizin Berlin, Corporate Member of Freie Universität Berlin, Humboldt-Universität zu Berlin, and Berlin Institute of Health, Berlin, Germany; Department of Vascular Surgery, Charité–Universitätsmedizin Berlin, Corporate Member of Freie Universität Berlin and Humboldt-Universität zu Berlin, Berlin, Germany; Department of Vascular Surgery, Charité–Universitätsmedizin Berlin, Corporate Member of Freie Universität Berlin and Humboldt-Universität zu Berlin, Berlin, Germany; Department of Vascular Surgery, Charité–Universitätsmedizin Berlin, Corporate Member of Freie Universität Berlin and Humboldt-Universität zu Berlin, Berlin, Germany; Center for Musculoskeletal Surgery, Charité–Universitätsmedizin Berlin, Corporate Member of Freie Universität Berlin, Humboldt-Universität zu Berlin, and Berlin Institute of Health, Berlin, Germany

**Keywords:** arterial vascular graft, infection, outcome, risk factor, treatment

## Abstract

**Background:**

Vascular graft infection (VGI) is a serious complication after implantation of arterial vascular grafts. Optimal surgical and pathogen-specific antimicrobial treatment regimens for VGI are largely unknown. We evaluated patients with arterial VGI according to onset, location, microbiological and imaging characteristics, and surgical and antimicrobial treatment and performed an outcome evaluation.

**Methods:**

Consecutive patients with VGI treated in 2 hospitals from 2010 through 2020 were retrospectively analyzed. Uniform definition criteria and standardized outcome evaluation were applied. Logistic regression was used for multiple analysis; survival analysis was performed with Kaplan-Meier analysis and a log-rank test.

**Results:**

Seventy-eight patients with VGI were included: 30 early-onset cases (<8 weeks after graft implantation) and 48 late-onset cases, involving 49 aortic and 29 peripheral grafts. The median time from initial implantation to diagnosis of VGI was significantly longer in aortic than peripheral VGIs (363 vs 56 days, *P* = .018). Late-onset VGI (odds ratio [OR], 7.3; *P* = .005) and the presence of surgical site infection/complication (OR, 8.21; *P* = .006) were independent risk factors for treatment failure. Surgical site infection/complication was associated with a higher risk for early-onset VGI (OR, 3.13; *P* = .040). Longer infection-free survival was observed in cases where the infected graft was surgically removed (*P* = .037).

**Conclusions:**

This study underlines the importance of timely diagnosis of VGI and preventing surgical site infections/complications at graft implantation. It highlights the complexity of infection eradication, especially for late-onset infections, and the importance of adequate antimicrobial and surgical treatment.

## Teaser

Aortic vascular graft infections (VGIs) were diagnosed significantly later than peripheral VGIs. Infection-free survival was significantly longer in cases with graft removal. Surgical site infection or complication at the time of initial graft implantation and a late onset of VGI are independent risk factors for treatment failure.

Vascular graft infection (VGI) is a serious complication occurring in 0.5% to 9% of implanted arterial vascular grafts [1–3]. Risk factors for infection include implantation-related factors (eg, bloodstream infection, surgical site infection, or groin incision) and patient-related factors (eg, renal impairment, higher age, or septic shock) [1, 2, 4–7]. Mortality rates range from 2.6% in peripheral VGIs to 8% in thoracic VGIs and up to 22% in patients with abdominal VGIs [1, 7, 8]. Severe complications during or after VGI treatment include development of fistula, anastomose insufficiency with acute bleeding, necessity for limb amputation due to ischemia, and persistent or recurring graft infection [8–11].

The management of VGI requires complex surgical revisions and prolonged antimicrobial treatment. Treatment decisions typically depend on expert opinion or individual clinical experiences. Uniform recommendations regarding the surgical and antimicrobial management of VGI are lacking, and the optimal treatment concepts are unknown. It is unclear when VGIs can be treated successfully with graft removal, considering risk of extensive surgical intervention potentially associated with high perioperative morbidity and mortality [8, 12, 13]. Thus, it is important to identify patient subpopulations and factors associated with beneficial or unfavorable impact on the treatment outcome of VGI.

In this study, we analyzed the characteristics of arterial VGI and identified risk factors for treatment failure of individual subgroups, including the impact of graft location, onset of infection, and type of antimicrobial and surgical treatment.

## METHODS

### Study Design

This retrospective observational study was performed at 2 tertiary cardiovascular surgery centers, cooperating with an interdisciplinary septic surgery unit. All patients from 1 January 2010 to 31 December 2020 coded with World Health Organization *ICD-10* codes Z95.88 (presence of other cardiac devices implants and grafts) and T82.7 (infection and inflammatory reaction due to other cardiac and vascular devices, implants, and grafts) were screened for study inclusion. Patients were included if the definition criteria for arterial VGI were fulfilled and the vascular graft was implanted at 1 of the 2 study centers. To keep the heterogeneity within the cohort at a minimum, we chose to exclude infections of endovascular grafts. Efforts are ongoing to analyze the endovascular graft infection cohort separately. Coronary bypass–associated infections were also excluded. This study was reviewed and approved by the institutional ethics committees of both study centers.

### Infection Definition

The definition criteria applied to arterial VGI in this study were established before the publication of the MAGIC criteria and are summarized in Supplementary Table 1 [8, 14]. This decision was made to avert further heterogeneity within the cohort by applying nonuniform definition criteria. VGI was confirmed if 1 definite criterion or 2 suggestive criteria were present at the time of diagnosis. Low-virulent pathogens such as coagulase-negative staphylococci, *Cutibacterium* spp, *Bacillus* spp, or *Corynebacterium* spp were considered pathogens if detected in at least 2 independent microbiological samples. Aortic infection included thoracic, abdominal, and thoracoabdominal VGIs, while peripheral VGIs included grafts of the peripheral arteries. VGIs were divided into early onset (diagnosed <8 weeks after initial implantation) and late onset (at ≥8 weeks after implantation). Extracted information included patient-specific data (demographics, comorbidities, underlying vascular disease), infection-specific data (diagnostic criteria, microbiology), surgical and antimicrobial treatment, and follow-up evaluation regarding treatment outcome.

### Outcome Evaluation

The follow-up period was defined as the period from the initial diagnosis of VGI to the last reported patient contact. An active follow-up with surviving patients was not pursued in this study.

The outcome was defined as treatment success if none of the following events occurred during the follow-up period: (1) reoperation at the vascular graft site; (2) infection-related death; or (3) persisting, recurring, or newly diagnosed VGI. Treatment failure was defined as the occurrence of any of these events. The infection-free survival interval was determined as the time from VGI diagnosis to the last reported patient contact at which a patient did not present with 1 of these events.

### Evaluation of Adequacy of Antimicrobial Therapy

Initial empiric antimicrobial treatment consisted of a broad-spectrum intravenous β-lactam antibiotic (eg, aminopenicillin with β-lactamase inhibitor or a cephalosporin of first, second, or fourth generation) in combination with a glycopeptide (eg, vancomycin, teicoplanin) or lipopeptide (daptomycin) antibiotic. In case of penicillin allergy, carbapenem (imipenem or meropenem) or fosfomycin was used to replace the penicillin derivative or cephalosporin.

Adequacy of treatment was evaluated according to antimicrobial adjustment to susceptibility testing (whether all pathogens were susceptible to at least 1 of the administered agents) and whether biofilm-active agents were used for sufficient treatment duration. Biofilm-active treatment was defined as administration of a rifampin-combination regimen for staphylococci, ciprofloxacin for gram-negative bacteria, ampicillin or amoxicillin for streptococci and enterococci, and metronidazole or clindamycin for anaerobic bacteria [6, 15–18]. If no biofilm-active antibiotics were available for drug-resistant pathogen strains, antimicrobial suppression for >1 year to lifelong was considered adequate treatment [19, 20]. The adequacy of antimicrobial treatment was evaluated by 2 independent infectious disease physicians who were blinded to the treatment outcome.

### Statistical Analysis

A sample size of 65 patients was estimated necessary for statistical analysis. A dropout rate of 10% to 15% was projected due to insufficient data, and a statistical power of 80% was estimated for 55 patients. SPSS (version 25.0; IBM) and RStudio (version 2023.06.1+524) were used for statistical analysis and plotting. For bivariate analysis, a chi-square test and Fisher exact test were applied for categorial variables and a Mann-Whitney *U* test for scaled variables. *P* < .050 was considered statistically significant. Multiple analysis was performed as logistic regression. Variables were selected forward according to statistical and clinical significance. We limited the number of variables for multiple analysis by applying *the rule of 10* [21]. Survival analysis was performed with Kaplan-Meier analysis. A log-rank test was used to compare survival rates of different patient subcohorts.

## RESULTS

### Patient Demographics and Comorbidities

Table 1 displays the demographic characteristics of the study cohort. Of 78 patients, 49 (62.8%) presented with aortic VGI and 29 (37.2%) with peripheral VGI. The anatomic location of aortic graft infections was thoracic in 23 cases, abdominal in 22 cases, and thoracoabdominal in 3 cases. The median patient age at diagnosis of VGI was 67.1 years. Most patients (95.4%) presented with 1 or more comorbidities, such as cardiovascular comorbidities, chronic renal insufficiency, previous thrombotic events, and diabetes mellitus.

**Table 1.** Demographic Characteristics of the Study's Patient Cohort

Characteristic	All Patients (n = 78)	Aortic VGI (n = 49)	Peripheral VGI (n = 29)	*P* Value
Age, y, median (range)	17.0–86.2 (67.1)	17.0–81.6 (66.6)	49.7–86.2 (70.6)	.349
Female sex	21 (26.9)	16 (32.7)	5 (17.2)	.138
Coexisting medical conditions				
Cardiovascular disease	72 (92.3)	44 (89.8)	28 (96.6)	.279
Chronic kidney failure	36 (64.2)	20 (40.8)	16 (55.2)	.219
Thrombotic events	32 (41.0)	15 (30.6)	17 (58.6)	.015
Diabetes mellitus	28 (35.9)	13 (26.5)	15 (51.7)	.025
Cardiac arrhythmia	23 (29.5)	16 (32.7)	7 (24.1)	.425
Active malignancy	21 (26.9)	10 (20.4)	11 (37.9)	.092
Obesity	19 (24.4)	16 (32.7)	3 (10.3)	.027
Valvular heart disease	18 (23.1)	14 (28.6)	4 (13.8)	.134
Heart failure	17 (21.8)	10 (20.4)	7 (24.1)	.700
Chronic obstructive pulmonary disease	16 (20.5)	7 (14.3)	9 (31.0)	.079
Other infectious focus at the time of VGI diagnosis^a^	9 (11.5)	7 (14.3)	2 (6.9)	.324
Factors present at initial graft implantation				
Groin incision	50 (64.1)	22 (44.9)	28 (96.6)	<.001
Perioperative infectious complication^b^	25 (33.3)	20 (43.5)	5 (17.2)	.019
Surgical site infection/complication^c^	32.1 (25)	28.6 (14)	34.5 (10)	.585
Emergency procedure	15 (19.2)	12 (24.5)	3 (10.3)	.126
Perioperative bacteremia	5 (6.4)	4 (8.2)	1 (3.4)	.646
Skin ulcers	4 (5.1)	1 (2.0)	3 (10.3)	.142

Data are presented as No. (%) unless otherwise indicated.

Abbreviation: VGI, vascular graft infection.

^a^Spondylodiscitis (n = 1), recurrent pyelonephritis (n = 1), septic arthritis in knee and ankle (n = 1), septic hip replacement (n = 1), decubitus (n = 1), osteomyelitis (n = 1), diabetic foot syndrome (n = 1), chronic vesicovaginal fistula (n = 1), and chronic sigmoiditis (n = 1).

^b^Perigraft infection (n = 6), sepsis/septic shock (n = 4), pneumonia (n = 3), peritonitis (n = 3), lymphatic fistula (n = 3), urogenital infection (n = 2), systemic inflammatory response syndrome (n = 2), systemic infection without focus (n = 2), mediastinitis (n = 1), abdominal abscess (n = 1), and limb infection (n = 1); n > 100% because several patients presented with more than 1 infectious complication.

^c^As defined by entry of T81.3 for surgical wound dehiscence or T81.4 for surgical site infection in the electronic patient chart.

Perioperative infectious complications during the index implantation of the later-infected graft were significantly more often observed in aortic VGIs than peripheral VGIs (*P* = .019), while surgical site infection or complication was observed at similar rates in both subgroups (*P* = .585). There was no significant correlation between the presence of surgical site infection/complication or perioperative infectious complication and the onset of VGI in the aortic subgroup (*P* = .129 and *P* = .535) or the peripheral subgroup (*P* = .069 and *P* = .060).

### Infection Characteristics

Table 2 summarizes the infection characteristics of the analyzed cohort. Thirty patients (38.5%) presented with early-onset VGI and 48 (61.5%) with late-onset VGI. The median time from initial implantation to diagnosis of VGI was 160 days and was significantly longer in aortic VGIs than peripheral VGIs (363 vs 56 days, *P* = .018). Among all patients, the most frequent clinical symptoms of VGI were discharge (n = 22) or pain (n = 22) at the surgical site and fever (n = 19). Aortic VGI more often manifested with systemic signs of infection such as fever (*P* < .001), whereas graft thrombosis or stenosis (*P* = .003) was more frequently observed in peripheral VGIs.

**Table 2.** Infection Characteristics of Vascular Graft Infection

Characteristic	All Patients (n = 78)	Aortic VGI (n = 49)	Peripheral VGI (n = 29)	*P* Value
Onset of infection				
Early-onset infection (<8 wk)	30 (38.5)	15 (30.6)	15 (51.7)	.064
Late-onset infection (≥8 wk)	48 (61.5)	34 (69.4)	14 (48.3)	.064
Time from initial graft implantation to infection diagnosis, d, median (range)	160 (1–7928)	363 (6–7928)	56 (1–2638)	.018
Clinical signs and symptoms at the time of initial diagnosis				
Fever	19 (24.4)	18 (36.7)	1 (3.4)	.001
Fatigue	7 (9.0)	7 (14.3)	0 (0.0)	.033
Surgical site erythema	23 (29.5)	8 (16.3)	15 (51.7)	.001
Surgical site discharge	22 (28.2)	12 (24.5)	10 (34.5)	.343
Local pain	22 (28.2)	11 (22.4)	11 (37.9)	.142
Graft thrombosis/stenosis	16 (20.5)	5 (10.2)	11 (37.9)	.003
Radiologic findings at the time of initial diagnosis				
Perigraft fluid	22 (28.2)	21 (42.9)	1 (3.4)	<.001
Perigraft air	15 (19.2)	12 (24.5)	3 (10.3)	.126
Perigraft abscess	15 (19.2)	11 (22.4)	4 (13.8)	.349
Graft thrombosis/stenosis	14 (17.9)	5 (10.2)	9 (31.0)	.021
Perigraft enhancement in PET/CT	12 (15.4)	12 (24.5)	0.0 (0)	.004
Aneurysm involving or surrounding the graft	10 (12.8)	6 (12.2)	13.8 (4)	.843
Perigraft lymphadenopathy	9 (11.5)	9 (18.4)	0 (0.0)	.014
Perigraft hematoma	7 (9.0)	6 (12.2)	1 (3.4)	.189
Anastomotic insufficiency/dehiscence/rupture	4 (5.1)	4 (8.2)	0 (0.0)	.291
Graft-organ fistula	2 (2.6)	2 (4.1)	0 (0.0)	.527

Data are presented as No. (%) unless otherwise indicated.

Abbreviations: CT, computed tomography; PET, positron emission tomography; VGI, vascular graft infection.

### Imaging Findings

Findings from radiologic and nuclear medical imaging are summarized in Table 2. Across all patients, visible perigraft fluid (n = 22), air (n = 15), and abscess (n = 15) surrounding the graft were the most frequently observed signs of VGI in diagnostic imaging. Supplementary Table 2 summarizes the rate of radiologic imaging suggestive of VGI. Computed tomography (CT) angiography and CT were the most frequently applied imaging methods (n = 50) and showed signs of VGI in 86.8%. If performed in aortic VGI, positron emission tomography/CT was positive for VGI in all cases, while all 3 cases of peripheral VGI that underwent such scans showed no enhancement (*P* = .038).

### Microbiological Data

Table 3 summarizes the cohort's microbiological characteristics of VGI analyzed in this study. Coagulase-negative staphylococci and *Staphylococcus aureus* were the most frequently isolated pathogens, followed by enterobacteria and enterococci. *Candida* spp were identified in 17 cases (21.8%): 12 aortic VGIs and 5 peripheral VGIs (*P* = .426). Strikingly, while the prevalence of polymicrobial VGIs was 41.6% across the total cohort, all cases but 1 (*Candida*) were polymicrobial (*P* < .001), matching the theory of microbiome translocation caused by surgery or trauma [22]. An increase of *Candida*-positive VGIs has been reported [19]. However, the influence of *Candida* as causative pathogens on the morbidity and mortality of patients with VGI remains unclear [23, 24]. In our cohort, we found no correlation between the presence of *Candida* and the location of the infected graft (*P* = .426) or the reported outcome (*P* = .132).

**Table 3.** Microbiological Characteristics

Pathogen	All Patients (n = 78)	Aortic VGI (n = 49)	Peripheral VGI (n = 29)	*P* Value
*Staphylococcus* spp	30 (38.5)	15 (31.3)	15 (51.7)	.074
Coagulase-negative staphylococci^a^	14 (18.0)	7 (14.3)	7 (24.1)	.273
*S aureus*	15 (19.2)	7 (14.3)	8 (27.6)	.163
Gram-negative bacteria ^b^	25 (32.1)	13 (27.1)	12 (41.1)	.194
*Enterococcus* spp	20 (25.6)	11 (22.9)	9 (31.0)	.431
*Candida* spp ^c^	17 (21.8)	12 (25.0)	5 (17.2)	.426
Anaerobes ^d^	8 (10.3)	6 (12.2)	2 (6.9)	.452
Streptococci	7 (9.0)	3 (6.3)	4 (13.8)	.265
*Pseudomonas* spp	7 (9.0)	2 (4.2)	5 (17.2)	.053
Drug-resistant pathogens ^e^	7 (9.0)	5 (10.4)	2 (6.9)	.603
Polymicrobial	32 (41.6)	19 (39.6)	13 (44.8)	.651
Culture negative	14 (18.2)	13 (27.1)	1 (3.4)	.009

Data are presented as No. (%).

Abbreviation: VGI, vascular graft infection.

^a^ *Staphylococcus epidermidis* (n = 10), *Staphylococcus hominis* (n = 2), *Staphylococcus haemolyticus* (n = 1), and *Staphylococcus lugdunensis* (n = 1).

^b^ *Escherichia coli* (n = 10), *Enterobacter cloacae* (n = 8), *Proteus mirabilis* (n = 6), *Klebsiella pneumoniae* (n = 5), *Klebsiella oxytoca* (n = 3), *Morganella morganii* (n = 3), *Proteus vulgaris* (n = 1), *Citrobacter braakii* (n = 1), *Citrobacter koseri* (n = 1), *Serratia marcescens* (n = 1), and *Hafnia alvei* (n = 1); n > 100% because several patients presented with more than 1 infectious complication.

^c^ *Candida albicans* (n = 12), *Candida glabrata* (n = 5), *Candida dubliniensis* (n = 2), and *Candida parapsilosis* (n = 1); n > 100% because several patients presented with more than 1 identified Candida strain.

^d^ *Bacteroides* spp (n = 5), *Peptoniphilus* spp (n = 2), *Finegoldia* spp (n = 2), *Prevotella* spp (n = 2), and *Anaerococcus* spp (n = 1); n > 100% because several patients presented with more than 1 infectious complication.

^e^As defined in the results regarding antimicrobial susceptibility testing, including vancomycin-resistant *Enterococcus faecium* (n = 3), methicillin-resistant *S aureus* (n = 1), multidrug-resistant *E coli* (n = 2), and multidrug-resistant *Morganella morganii* (n = 1).

Of 14 culture-negative infections, 1 was located peripherally, and 13 were aortic VGIs (*P* = .009).

Multidrug-resistant pathogens were found in <10% of patients. Patients with polymicrobial infections received inadequate antimicrobial treatment significantly more often than those with monomicrobial infections (54.1% and 30.0%, *P* = .032).

Supplementary Table 3 summarizes the type of microbiological sample in which the pathogen was isolated. Intraoperative samples and explanted graft material grew pathogens significantly more often in peripheral VGIs than aortic VGIs (*P* = .005 and *P* = .036, respectively).

### Antimicrobial Treatment

In our cohort, all patients received antimicrobial treatment. The median time of initial antimicrobial therapy was 50 days (range, 12–189). A biofilm-active antibiotic was included in 28 cases (41.2%), and suppressive antimicrobial treatment was administered in 27 cases (36.5%). Appropriate biofilm-active agents and suppressive antimicrobial treatment were found in antimicrobial adequately treated patients significantly more often (both *P* < .001) but did not affect the outcome (*P* = .486 and *P* = .844, respectively). New pathogens during treatment occurred in 18 cases (25.7%) and significantly more often in patients with inadequate antimicrobial therapy (39.4% and 13.5%, *P* = .013). Overall adequate antimicrobial treatment was observed in only 51.3% of the cohort. Inadequately treated infections consisted of 13 monomicrobial, 20 polymicrobial, and 4 culture-negative infections. The proportions of detected pathogens are similar to those observed in the total cohort, with staphylococci being the most frequently detected group. Of cases with inadequate antimicrobial treatment (n = 38), 20 patients received only 1 antimicrobial agent in the initial antimicrobial regimen.

For the primary end point of infection-free survival, 50% of all patients with adequate antimicrobial treatment later showed treatment success, as opposed to only 39.5% (*P* = .350) of patients with inadequate antimicrobial therapy.

### Surgical Treatment

**Figure 1.**
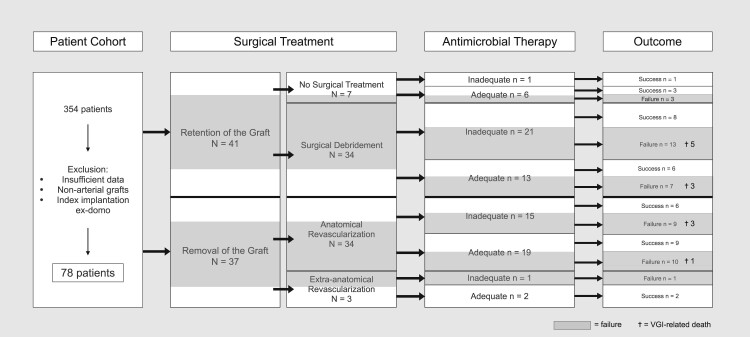
Study cohort according to treatment mode and outcome. VGI, vascular graft infection.

Figure 1 shows the study cohort according to surgical treatment mode and outcome. In the presented cohort, 37 patients underwent graft removal (34 anatomic and 3 extra-anatomic revascularizations); 34 patients received graft-retaining surgery with debridement of the infected field; and 7 patients did not receive any surgical intervention for VGI. Among surgically treated patients, 56.3% experienced peri- or postoperative complications within the first 10 days of surgery: significant bleeding (n = 13), peripheral ischemia (n = 8), and graft thrombosis (n = 6). Infectious complications occurred in 14 cases (19.7%), of which pneumonia was the most frequent (n = 4). Furthermore, 16 patients (22.5%) experienced major systemic complications, such as respiratory insufficiency, acute kidney failure, or hemodynamic decompensation. Additional surgery was necessary in 22 cases (28.2%), of which 17 (22.1%) underwent consecutive amputation of the leg. Peripheral VGIs required additional surgical interventions significantly more often than aortic VGIs (75.9% vs 28.6%, *P* = .002).

Postoperatively, patients with inadequate antimicrobial therapy showed significantly more infectious complications (*P* = .010), more often required escalation of surgical treatment (*P* = .008) and amputation (*P* = .002), and had a higher total number of surgical procedures (*P* = .013).

### Outcome

The median time from VGI diagnosis to last patient contact or death was 6.87 months (range, 0.13–55.89). Thirty-five patients (44.9%) were infection-free at the time of last contact. Overall, only 44.9% of the cohort (n = 35) had successful eradication of VGI. This number was even lower in the subgroup of late-onset infections (35.4%, *P* = .034).

Across the whole cohort, 31 patients (39.74%) presented with persisting infection (n = 15) or reinfection (n = 16) during follow-up. Out of 16 documented reinfections, 10 occurred in the cohort with inadequate antimicrobial treatment (*P* = .072). Infection-related adverse events were reported during follow-up in 24.4%: septic shock (n = 7), anastomotic bleeding (n = 4), surgical site infection (n = 4), graft thrombosis (n = 3), graft-organ fistula (n = 3), seroma (n = 3), bowel ischemia or perforation (n = 2), organ failure (n = 2), compartment syndrome (n = 1), amputation (n = 1), and peripheral edema (n = 1). The rate of these events was similar in aortic (24.5%) and peripheral (24.1%) VGIs but was almost twice as high in patients who received inadequate antimicrobial treatment as compared with those receiving adequate treatment (31.6% and 17.5%, *P* = .148).

During follow-up, 18 deaths (23.1%) were reported, including 12 VGI-related deaths. Although this was a nonsignificant trend, the rate of VGI-related deaths was twice as high in patients with inadequate antimicrobial therapy (21.1% and 10.0%, *P* = .176).

**Figure 2.**
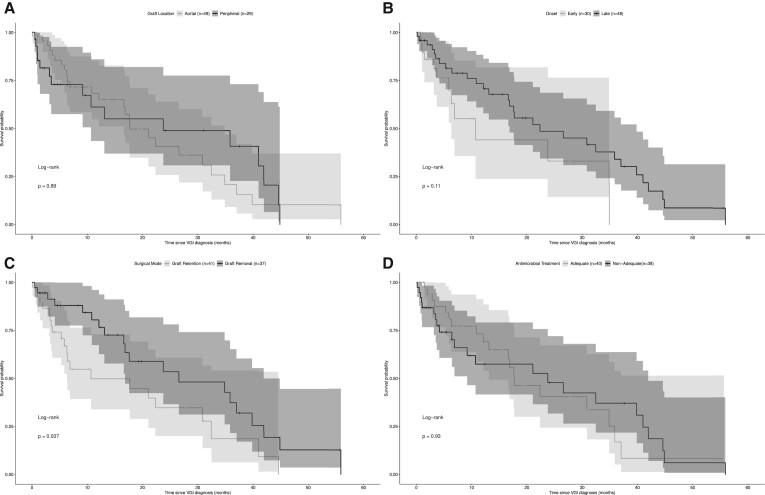
Kaplan-Meier analysis of infection-free survival according to (*A*) graft location, (*B*) onset, (*C*) surgical mode, and (*D*) adequacy of antimicrobial therapy. Only the surgical mode (ie, removal of the infected graft) had a positive significant impact on the infection-free survival (*P* = .037). Shaded area indicates 95% CI. VGI, vascular graft infection.

Figure 2 visualizes the Kaplan-Meier analysis of infection-free survival according to location of the infected graft, VGI onset, surgical mode, and adequacy of antimicrobial treatment. Onset, graft status, and adequacy of antimicrobial treatment did not have an impact on the infection-free survival of patients in our cohort. Only graft removal was associated with longer infection-free survival (Figure 2*C*; median [95% CI], 10.68 months [0–25.75] vs 26.61 months [4.34–48.89]; *P* = .037).

To identify independent risk factors for treatment failure, we further performed a logistic regression. In this model applied to the whole study cohort, we identified surgical site infection or complication after the initial graft implantation (odds ratio, 8.2; 95% CI, 1.86–36.36; *P* = .006) and late-onset VGI (odds ratio, 7.3; 95% CI, 1.83–28.92; *P* = .005) as independent risk factors for treatment failure.

## DISCUSSION

In this study, we present 78 retrospectively analyzed cases of arterial VGI according to anatomic location, onset, treatment, and outcome of VGI. Several of our findings are relevant to the clinical management of VGI and may affect the treatment outcome: (1) only about half of all analyzed patients received adequate antimicrobial therapy; (2) patients with removal of the infected graft had a longer infection-free survival; (3) aortic VGIs more often presented as late-onset infections than peripheral VGIs; and (4) late onset of VGI and surgical site infection or complication at the initial graft implantation are independent risk factors for treatment failure in VGI.

Only about half of the analyzed cohort received overall adequate antimicrobial therapy. Although this number is similar to previously reported cohorts, it is lower than expected [25]. Possible reasons were the use of single-agent antimicrobial regiments, limited spectrum therapy in polymicrobial infections, and the lack of antifungal agents in empirical antimicrobial therapy. Due to increasing numbers of fungal VGI, the inclusion of an antifungal agent in the empirical treatment, especially for intracavitary VGI, is recommended [19, 26].

Patients with inadequate antimicrobial therapy showed higher rates of new pathogen growth during treatment and persisting infection and reinfection during follow-up, subsequently resulting in treatment failure in most cases. This subgroup also more frequently required surgical escalation, which in this highly comorbid cohort poses a higher risk for complications itself. To prevent the growth of new or untargeted pathogens, patients should receive broad initial empirical antimicrobial treatment, which can be de-escalated rather than escalated after microbiological testing. Like most published retrospective studies addressing VGI treatment, we are not able to provide a data-based recommendation for the exact duration of antimicrobial treatment. To overcome this shortcoming, prospective studies need to be conducted evaluating the exact duration of antimicrobial therapy for VGI.

In Kaplan-Meier analysis, patients who had their infected grafts removed showed significantly longer infection-free survival as compared with patients who did not have their grafts removed, highlighting the impact of surgical treatment decision making in the management of VGI. Similar results have been reported presenting favoring outcomes for patients with graft removal [10–12]. A possible limitation to this result is the proportion of patients in the graft retention cohort not consenting to or being too unfit for extensive surgery, including 7 cases that did not receive any surgical therapy for VGI. Even though we found no significant differences in medical comorbidities or age between patients with and without graft removal in our cohort, factors not documented in this study, such as nonconsent to surgery, general frailty, altered mental state, or level of care, could imply a higher rate of patients likely to have bad outcomes without regard to the surgical regimen in the graft retention cohort. In these cases, previous studies have presented good results from less-invasive graft-retaining surgery, also in combination with prolonged antimicrobial therapy [8, 25, 27, 28]. Based on these results, a less-invasive graft-retaining surgical approach can be individually discussed with patients of elevated surgical risk, inoperability, or wish for less invasive surgery. However, our results strongly suggest that if operability is granted, removal of the infected graft should be the standard surgical approach in VGI.

In our study, patients with peripheral VGIs had an earlier onset of infection than patients with aortic VGIs. This finding is concordant with results from a previously published study [29]. While a cause could be the more subtle clinical appearance of aortic VGIs possibly prolonging the time to diagnosis, further research on the reasons for a later onset of aortic VGI needs to be conducted [30]. Our results show good radiologic and nuclear imaging accuracy for the detection of aortic VGIs. To improve the time to diagnosis of aortic VGI, we therefore suggest radiologic and nuclear medical imaging alongside microbiological culture as recommended by the MAGIC consortium to rule out or confirm VGI in patients with an inlaying aortic vascular graft presenting with a fever of unclear focus [31, 32].

Surgical site infection has been previously determined as an independent risk factor for the development of VGI [1, 2, 4]. To our knowledge, our study is the first to also determine surgical site infection or complication as a risk factor for treatment failure of VGI. Thus, reducing the number of surgical site infections and other surgical site complications at the initial implantation of vascular grafts is important to improve the management of VGI.

Another independent risk factor for treatment failure was a late onset of VGI, which in our study was surprisingly not influenced by surgical or antimicrobial treatment status. Thus, other influences must be considered. In nonsignificant trends, patients with late-onset VGI presented with a higher median age at diagnosis, twice as many VGI-related deaths, and 3 times as many reinfections as in early-onset VGIs. Age >70 years at diagnosis has been described as a risk factor for in-hospital mortality of patients with aortic VGIs [5, 7]. Another possible factor for treatment failure in the late-onset cohort is the insufficient eradication of mature biofilm. Mature biofilm is less susceptible to common antimicrobial agents and shows increased production when exposed to high shear forces and fluid flow rates, as can be found in the arterial vascular system [33–39]. In our study, neither the inclusion of a biofilm-active agent nor overall adequate antimicrobial therapy had a significant impact on the outcome. Since scientific evidence for biofilm activity of antimicrobial agents other than rifampin and fluroquinolones is controversial, other approaches of biofilm-active treatment should be considered [17, 18]. Multimodal and novel approaches for biofilm eradication, including the use of bacteriophages, matrix-degrading enzymes, and improved graft material, should be considered in the treatment, especially for late-onset VGIs [37, 40–44]. Besides sufficient biofilm eradication, further research needs to be conducted focusing on other causes for poor outcome of late-onset VGI.

In our study, reinfections or persisting infections during follow-up occurred in 31 cases, majorly contributing to the relatively large proportion of negative outcomes. To address this shortcoming, more studies are needed to establish validated follow-up regimes after VGI, such as further evaluation of the impact of nuclear imaging as a diagnostic parameter for therapy and relapse monitoring [45–47].

Our study is limited by its retrospective design, dependency on documented data in the hospital's electronic patient record, the inclusion of 2 hospitals with possible varying internal practices, the limited number of patients, and the limited median duration of follow-up. However, we applied uniform definition criteria for VGI, and both institutions followed the same treatment recommendations and were consulted by the same infectious disease consultation service. Even though the absolute number of patients in our study is small and limits detailed statistical analysis, we present one of the largest cohorts of patients with arterial VGI. We were able to assess the impact of location, onset, and treatment of infection, as well as new risk factors for treatment failure of VGI, and we present new findings that will ideally help to improve the challenging management of VGI in the future. Our results also stress the need for prospective study designs to further optimize the diagnostic workup and surgical and antimicrobial treatment of VGI.

## Supplementary Material

ofae271_Supplementary_Data

## Supplementary Data

Supplementary materials are available at *Open Forum Infectious Diseases* online. Consisting of data provided by the authors to benefit the reader, the posted materials are not copyedited and are the sole responsibility of the authors, so questions or comments should be addressed to the corresponding author.

## Notes

***Author contributions.*** L. S. and A. T. designed the study. L. S. was responsible for data acquisition and performed the reported analysis. L. S. wrote the original draft and was responsible for reviewing literature. D. M. and A. T. evaluated the adequacy of antimicrobial treatment. S. O., M. S., and A. G. revised the manuscript and provided expertise on the vascular surgery aspects. All others revised and edited the manuscript. L. S. and A. T. had full access to the original data and take full responsibility for the integrity of the data and the accuracy of performed analyses.

***Data availability***. The original raw data of this study are available upon request to the authors.

***Patient consent statement.*** This study (EA2/233/20) was evaluated and approved by the institutional ethics committee, following the 1964 Declaration of Helsinki. The ethics committee waived the need for patient informed consent due to the retrospective character of the study.

***Financial support***. This work was supported by the German Research Foundation; and the institutional fund for open-access publication of scientifc work.
