# Supplementary material for: Characteristics and Outcome of Vascular Graft Infections: A Risk Factor and Survival Analysis
Source: Open Forum Infect Dis. 2024 May 13;11(6):ofae271. doi: 10.1093/ofid/ofae271 (PMC11167665; doi:10.1093/ofid/ofae271)
Supplement: ofae271_Supplementary_Data [file ofae271_supplementary_data.docx]

**SUPPLEMENTARY MATERIAL**

**Supplementary Table S1** Definition Criteria for Vascular Graft Infection

| Category | Definite Criteria | Suggestive Criteria |
| --- | --- | --- |
| Clinical Signs | - Purulent secretion at surgical site - Perigraft sinus tract or abscess - Wound dehiscence or graft-surface contact - Aortoenteric fistula - Intraoperative purulence | - Gastrointestinal bleeding |
| Histopathology | - Inflammation of perigraft tissue |  |
| Microbiology | - Pathogen-positive perigraft or prosthetic tissue - Pathogen-positive perigraft fluid - Pathogen-positive deep intraoperative perigraft swab - Pathogen-positive sonication of vascular graft - Two or more positive blood cultures without any other apparent infectious focus | - Single positive blood culture without any other apparent infectious focus |
| Radiology | - Perigraft abscess - Lack of graft incorporation into surrounding tissue | - Perigraft air or fluid more than 8 weeks after initial graft implantation |

| Modality | Performed | Positive | Negative |
| --- | --- | --- | --- |
| Computer tomography (CT) | 9 (11.5) | 7 (77.8) | 2 (22.2) |
| CT-Angiography | 50 (64.1) | 43 (86.0) | 7 (14.0) |
| Magnetic resonance imaging | 1 (1.3) | 1 (100) | 0 (0.0) |
| Sonography | 30 (38.5) | 17 (56.7) | 13 (43.3) |
| Positron emission tomography (PET)-CT | 15 (19.2) | 12 (80.0) | 3 (20.0) |
| Angiogram | 10 (12.8) | 7 (70.0) | 3 (30.0) |

**Supplementary Table S2** Radiological and nuclear medical imaging modalities and yielded positive/negative results for the overall cohort.

Data are in No. (%) of patients unless otherwise indicated.

**Supplementary Table S3** Microbiological Diagnostic Tests

| Diagnostic | Performed | Positive | Negative |
| --- | --- | --- | --- |
| Blood cultures | 51 (65.4) | 26 (51.0) | 25 (49.0) |
| Aspirated perigraft fluid | 18 (23.1) | 10 (55.6) | 8 (44.4) |
| Perigraft tissue | 34 (43.6) | 23 (67.6) | 11 (32.4) |
| Graft material | 36 (46.2) | 20 (55.6) | 16 (44.4) |
| Sonication | 5 (6.4) | 0 (0.0) | 5 (100) |
| intraoperative swab | 68 (87.2) | 53 (81.5) | 15 (22.1) |

Data are in No. (%) of patients unless otherwise indicated
